# Surgical Site Infection in Thoracic Surgery Is Not Associated With Perioperative Hypothermia

**DOI:** 10.7759/cureus.26427

**Published:** 2022-06-29

**Authors:** Albert P Nguyen, Minh Tran, Swapnil Khoche, Rodney A Gabriel, Ulrich Schmidt

**Affiliations:** 1 Division of Critical Care Medicine, Providence Santa Rosa Memorial Hospital, Santa Rosa, USA; 2 Anesthesiology, University of California San Diego Health, La Jolla, USA

**Keywords:** performance measure, surgical care improvement project, thoracic surgery, hypothermia, surgical site infection

## Abstract

Introduction: The Surgical Care Improvement Project (SCIP) added the SCIP-Inf-10 measure to mandate that all surgical patients have perioperative temperature management to reduce surgical site infection. While the basis of this measure originated in colorectal surgery, we hypothesized that this would also apply to thoracic surgery patients.

Methods: This was a retrospective single-center pilot study reviewing two years of thoracic surgery cases for the incidence and duration of hypothermia during the operation and surgical site infection occurring within 30 days. Hypothermia was defined as a core temperature of < 36° C.

Results: A total of 317 patients were included in the study. Sixty-two percent of patients were identified as hypothermic. The average intraoperative temperature was 35.4°C ± 0.8°C in the hypothermic group and 36.4°C ± 0.3°C in the normothermic group. There were four surgical site infections in the study with three cases from the <36°C group (p = 1). There was no difference in average post-anesthesia care unit length of stay between the groups. The average hospital length of stay was 5.5 ± 5.2 days for the hypothermic group and 8.6 ± 12.8 days for the normothermic group (p=0.0024).

Conclusion: Perioperative hypothermia was common in thoracic surgery and did not have a negative impact on surgical site infection.

## Introduction

Surgical site infection (SSI) has been attributed to longer hospitalizations, increased morbidity and mortality, and higher healthcare costs [1,2]. The Surgical Care Improvement Project (SCIP) was organized with the goal of reducing surgical infection by developing a series of standardized quality improvement measures that could be applied nationally [1]. One such measure was the Surgical Care Improvement Project- Infection (SCIP-INF) 7 which required reporting of the use of an active warming device and normothermia immediately after colorectal operations. This measure was later replaced by SCIP-INF 10, which mandated reporting of an active warming device for all surgeries. The foundation for these measures was a study by Kurz et al., in which the authors reported an association between intraoperative hypothermia and an increase in surgical site infection in the colorectal surgery population [3]. In this pilot study, we focused on intraoperative hypothermia and the rate of postoperative SSI in thoracic surgery patients at a single institution. Previously reported surgical site infection for thoracic surgery has ranged from 0.4 to 11.4% [4,5]. As hypothermia was associated with SSI in colorectal surgery, we hypothesized that hypothermia would also be associated with a higher rate of SSI in a thoracic surgical patient.

## Materials and methods

This retrospective study was approved by the University of California San Diego (UCSD) Institutional Review Board (IRB) (approval number 161792). The UCSD cardiothoracic surgery registry was used to identify patients undergoing thoracic surgical procedures from January 2014 to December 2016. Thoracic surgical procedures were defined as those patients undergoing a thoracotomy and/or video-assisted thoracoscopic surgery (VATS). Patients excluded from this study included those undergoing surgery for lung transplantation, decortication, surgical debridement, surgery confined to the chest wall, and patients with an active infection. Patient data were abstracted from our institution’s electronic medical record (Epic Systems Corporation, Madison, WI). This was a retrospective cohort study aimed to assess an association between intraoperative core temperature measurement and postoperative SSI. The primary outcome measure was the occurrence of postoperative SSI. SSI was defined using the Center for Disease Control and Prevention’s criteria (Table 1).

**Table 1:** Surgical Wound Classifications as Defined by the American College of Surgeons National Surgical Quality Improvement Program

Wound Classification	Definition
Clean	These are uninfected operative wounds in which no inflammation is encountered and the respiratory, alimentary, genital, or uninfected urinary tracts are not entered.
Clean/Contaminated	These are operative wounds in which the respiratory, alimentary, genital, or urinary tract is entered under controlled conditions and without unusual contamination.
Contaminated	These include open, fresh, accidental wounds, operations with major breaks in sterile technique or gross spillage from the gastrointestinal tract, and incisions in which acute, non-purulent inflammation is encountered.
Dirty	These include old traumatic wounds with retained devitalized tissue and those that involve existing clinical infection or perforated viscera.

Positive identification of SSI was accomplished by reviewing each patient’s medical record for the first 30 days post-procedure. Their post-operative clinic visit, hospitalization, and/or emergency room visit were assessed for documentation of surgical site infection, purulence, and/or the initiation of antibiotics related to SSI. The primary exposure variable was intraoperative core temperature measurement. Intraoperative core temperature was measured either by an esophageal or bladder temperature probe and was recorded every five minutes in the electronic medical record. Secondary outcomes measured included the intraoperative use of a forced-air warming device, initial post-anesthesia care unit (PACU) temperature measured within five minutes of arrival by a temporal thermometer, PACU length of stay as determined by modified Aldrete score, hospital length of stay, and in-hospital mortality. 

Statistics

Patients were grouped into the following categories: (1) intraoperative core temperature of ≥ 36°C, (2) intraoperative core temperature of < 36°C, and (3) no temperature recorded. Differences in categorical variables were assessed with Fisher exact test and for continuous variables, the differences in mean were measured by the Student t-test. For both groups, a p-value < 0.05 was considered to be statistically significant. 

## Results

A total of 317 patients met the predefined criteria and were included in this analysis. From the data abstracted, two cohorts were created. A third group did not have recorded intraoperative temperature measurements (n = 57) and was analyzed separately. The baseline characteristics of these three groups are presented in Table 2.

**Table 2:** Baseline Characteristics kg/m2 = Kilograms per meter squared.

Comorbidity	Temperature < 36°C (n = 230)	Temperature ≥ 36°C (n = 87)	No Temperature Recorded (n = 57)
Thoracotomy (n)	23	17, p = 0.035	7, p = 0.36
Average Age (years)	58.9 ± 16.2	56.2 ± 16.1	56.9 ± 16
Average BMI (kg/m^2^)	26.5 ± 9	27.8 ± 6.9	26.6 ± 7.2
Active Smoking (n)	25	9, p = 1.00	8, p = 0.33
Diabetes Mellitus (n)	33	24, p = 0.009	8, p = 1.00
Coronary Artery Disease (n)	62	29, p = 0.26	17, p = 0.74
Chronic Obstructive Pulmonary Disease (n)	43	23, p = 0.63	7, p = 0.33
Chronic Kidney Disease (n)	16	9, p = 0.35	3, p = 0.77
Malignancy (n)	171	43, p = 0.001	42, p =1.00
Peripheral Vascular Disease (n)	5	7, p = 0.02	1, p = 1.00
Preoperative Steroid Use (n)	3	8, p = 0.002	3, p = 0.09
Bladder Temperature Use (n)	8	3, p = 1.00	1, p = 0.69
Esophageal Temperature Use (n)	222	84, p = 1.00	15, p < 0.01
Forced Air Warming Device (n)	213	78, p = 0.37	45, p < 0.01
Postoperative Chest Tube (n)	210	75, p = 0.66	47, p = 0.09
Postoperative Mechanical Ventilation (n)	24	18, p = 0.025	8, p = 0.48

The first cohort was defined as patients with an intraoperative core temperature of > 36°C (n = 87). In this group, the average intraoperative core temperature was 36.4°C ± 0.3°C with a recorded nadir temperature of 35.9°C ± 0.6°C. The second cohort was of those patients who had a core intraoperative temperature of < 36°C (n = 230). The average intraoperative core temperature was 35.1°C ± 0.6°C with a nadir of 34.7°C ± 0.8°C. The duration of hypothermia for each group is reported in Table 3. A patient warming device was used in 95.1% of the patients in the > 36°C group, 93.2% of patients in the < 36°C, and 82.5% of the no temperature group. 

**Table 3:** Outcome Measures for Intra-op T ≥ 36°C and Intra-op T <36°C. Surgical Site Infections (SSI) within 30 days, EBL = estimated blood loss, LOS= length of stay, MAC = minimum alveolar concentration, MV = mechanical ventilation, mmHg = millimeters of mercury, ml = milliliter, PACU = post anesthesia care unit, OLV = one lung ventilation

Perioperative Outcomes	Intraop T ≥36°C N= 82		Intraop T<36°C N= 230	P value
Surgical Site Infections (n)	1	3		1.00
Average Surgical Time (minutes)	129.3 ± 86.5	136± 63.7		0.52
Average Temperature (Celsius)	36.4°C ± 0.3	35.1°C ± 0.6		< 0.001
Average Nadir Temperature (Celsius)	35.9°C ± 0.6	34.7°C ± 0.8		< 0.001
Average Time < 36 C (minutes)	5.79 ± 15.1	137.1 ± 66.2		< 0.001
Average SpO_2_ on OLV (%)	99 ± 3.8	98.9 ± 2.4		0.19
Average Intraoperative MAP (mmHg)	78.7 ± 12.9	78.9 ± 14.3		0.23
Average Intraoperative MAC	0.89 ± 0.13	0.87 ± 0.12		0.62
EBL Thoracotomy (ml)	198.2 ± 469.1	131.3 ± 128.6		0.51
EBL Video Assisted Thorascopy (ml)	73.8 ± 208	45.4 ± 66.6		0.08
PACU Arrival Temp (Celsius)	36.5 ± 0.44	36.2 ± 0.41		0.0024
PACU LOS (mins)	218± 115	227 ± 115		0.54
Hospital LOS (days)	8.6 ± 12.8	5.5 ± 8.0		0.0024
Postoperative MV (n)	18	24		0.025
Postoperative Chest Tube (n)	75	210		1.00
Mortality (n)	0	1		1.00

The total number of patients with an SSI was 4 (1.1%). Three of these patients were in the temperature <36°C group and the other was in the temperature > 36°C group. There was no significant difference in estimated blood loss between the < 36°C and the >36°C. The duration of time where the core temperature was < 36°C is reported in Table 3. The > 36°C had a significantly higher initial temperature measurement upon PACU arrival compared to the < 36°C. The PACU length of stay was similar and the hospital length of stay for thoracotomies was similar between the < 36°C and the >36°C. For those patients who had undergone a VATS procedure, the < 36°C group had a significantly shorter length of stay compared to the >36°C (Table 3). 

Description of patients with surgical site infection

In the > 36°C group, patient #1 was a 55-year-old woman with a past medical history significant for coronary artery disease who underwent a right-sided thoracotomy with a right upper and lower lobectomies for adenocarcinoma. Preoperatively, the patient had not been on antibiotics or steroids. The patient did not require an intraoperative blood transfusion during the operation and had received perioperative antibiotics. Active warming blanket had been applied during surgery. The patient did not require postoperative mechanical ventilation and was discharged home on post-operation day (POD) five. The patient was seen in the clinic one week later and purulent drainage from the surgical site was seen. She was admitted to the hospital for incision and drainage of her infected site and was discharged home on antibiotics. 

The < 36°C group had three patients with surgical site infection. Patient #1 was a 55-year-old woman with no past medical history who had undergone a VATS with a right lower lobe wedge resection for a pulmonary node. The patient had not been taking systemic steroids or antibiotics preoperatively. The patient had received perioperative antibiotics, an active warming blanket had been placed on the patient, and she did not require an intraoperative blood transfusion. The patient was extubated post-procedure and was discharged home on POD# 2. The patient was seen in the clinic 20 days later and the incision site was noted to be erythematous, indurated, and tender without drainage. The patient was started on a 10-day course of antibiotics without the need for incision and drainage.

Patient # 2 was a 60-year-old woman with a history of hepatitis C and chronic obstructive pulmonary disease who underwent a VATS with right lower lobectomy for adenocarcinoma. The patient had received perioperative antibiotics, an active warming blanket was placed on the patient, and no intraoperative blood transfusions were required. She was extubated at the end of the procedure. She was discharged home on POD # 7 due to difficulty in pain control. The patient was seen in the clinic 10 days post-discharge. The surgical site, on examination, was erythematous with purulent drainage. The patient was started on antibiotics and did not require admission for surgical intervention.

Patient #3 was a 70-year-old man with a history of coronary artery disease who had undergone a VATS with right upper lobectomy for adenocarcinoma. He had not been on steroids or antibiotics preoperatively. Intraoperatively, the patient had received prophylactic antibiotics and had an active warming blanket applied. There was no blood transfusion administered during the operation and the patient was extubated at its conclusion. The patient was discharged home on POD# 3. Fifteen days post-discharge, the patient presented to the emergency department for purulent discharge and erythema at his surgical site. He was admitted to the hospital for surgical debridement of his incision site and received a 14-day course of antibiotics.

## Discussion

In this retrospective cohort study, we did not find an association between intraoperative temperature and postoperative SSI in patients who underwent thoracic surgery. Intraoperative hypothermia is postulated to predispose patients to SSI. The cold temperature is thought to induce peripheral vasoconstriction that impedes tissue oxygenation, collagen deposition, and oxidative killing of microbes by neutrophils [6]. We, however, did not observe a difference in the rate of SSI between patients who were normothermic intraoperatively and those who were hypothermic.

In our study, most patients had intraoperative hypothermia (62.3%) despite nearly all of them having an active warming device applied (97%). In the > 36°C group, the duration of hypothermia was brief and occurred post-induction of general anesthesia, similar to reported studies [7,8]. In the < 36°C group, their duration of hypothermia was significantly longer despite active warming measures. Lenhardt et al. reported that mild intraoperative hypothermia, defined as a temperature of approximately 34.5°C, was associated with a delayed PACU length of stay (LOS) of up to 90 mins compared to normothermic patients [9]. We found no difference in the duration of PACU stay between hypothermic and normothermic patients. The normothermic group had a significantly longer hospital stay compared to the hypothermic group. The normothermic group also had significantly higher rates of postoperative mechanical ventilation. The increase in postoperative mechanical ventilation in this group is likely attributable to the higher number of thoracotomies than due to temperature.

While both colorectal and thoracic surgical procedures share cancer resection as a surgical indication, indications for colorectal surgery also include diverticulitis, inflammatory bowel disease, and correction of fistulas; all disease processes that expose the patient to bowel contamination. Furthermore, patients with gastrointestinal diseases are predisposed to chronic malnutrition and may be on immunomodulators. These are factors that lead to SSI by impairing wound healing and lowering immune defense [10,11]. Compared to thoracic surgery, the surgical wound classification, a risk assessment model in use since 1964 (Table 1), has placed colorectal procedures in a higher category depending on the surgical location, disease type, and spillage from the alimentary tract [12]. Given the difference between the two groups, there may be a misapplication of interventions in the thoracic surgery population.

In 2009, SCIP-INF 7 was replaced with SCIP-INF 10, requiring reporting of active warming for normothermia immediately after all operations, however, the only prospective trial study supporting this process measure was by Kurtz et al. in 1996 [2,6]. This SCIP measure assumes that an endpoint of a normothermic core temperature in the PACU is a result of normothermia in the operating room. Our study demonstrates that while most patients are hypothermic in the operating room, 78% of these patients arrived at the PACU normothermic. These results are in line with other studies involving orthopedic and non-cardiothoracic surgical cases [2,13]. More recent studies have questioned whether normothermia is responsible for lowering surgical site infection. In a matched control study involving non-colorectal abdominal patients, Lehtinen et al. found no independent association between perioperative normothermia and SSI. Instead, diabetes, open surgical approach, and surgical complexity were independent risk factors [6]. Baucom et al., in a retrospective cohort study involving surgical colectomies, did not observe a difference in rates of SSI between hypothermic and normothermic patients. What was unique to this study was that they examined the incidence of hypothermia, duration of hypothermia, and nadir temperature. However, it should be noted that this study excluded patients with inflammatory bowel disease and planned ostomies [14]. Brown et al. did not find intraoperative hypothermia to be a contributing factor to SSI, particularly in patients with a Class I clean wound site [2]. 

Clearly, the goals of surgical care are to improve survival rates and reduce morbidity. The concern is whether we are employing the correct performance measures to achieve these goals. For instance, based on the DECREASE trial, SCIP measure, SCIP-Card-2 was created. It required the administration of perioperative beta-blockers for all surgical patients who regularly took beta-blockers. Later evidence showed that perioperative beta-blockade resulted in reduced acute myocardial infarctions and ischemia but at the risk of increased all‑cause mortality and cerebrovascular events [15]. These process measures, while attempting to improve patient outcomes, may instead be harming patients. The use of a forced warm air device did not cause harm in our study, though there have been reports of it leading to contamination of the sterile surgical environments [16].

The retrospective nature of the study has certain limitations. The accuracy of the data may be limited by an incomplete recording of the information. It is possible that some patients may have had warming devices used, but not recorded. The incidence of surgical site infection was dependent on documentation in the chart and that the patient received follow-up care at UCSD and not elsewhere. All patients in this study, unless still admitted, were seen in the thoracic surgery clinic within four weeks of their operation. The strength of this study is that it makes use of the intraoperative medical record. This allows for automated data collection and did not rely on hand-collected data. Though this may be a single-center study, there is strength in the fact that the medical center employs only two thoracic surgeons. This reduces practice pattern variability and the risk of surgeons as a risk factor for SSI [17].

## Conclusions

Relative hypothermia in thoracic surgery is common. The degree of hypothermia and its duration does not appear to affect PACU LOS or SSI in this population. In this study, the use of an active warming device does not affect patient outcomes, nor does it confer harm. We advocate constant scrutiny of performance measures for patient outcomes and should strive to modify them based on the best available data.
